# Poorly differentiated clusters with larger extents have a greater impact on survival: a semi-quantitative pathological evaluation for 239 patients with non-mucinous pT2-3 colorectal carcinoma

**DOI:** 10.1186/s12957-015-0550-5

**Published:** 2015-04-08

**Authors:** Osamu Kinoshita, Mitsuo Kishimoto, Yasutoshi Murayama, Satoru Yasukawa, Eiichi Konishi, Eigo Otsuji, Akio Yanagisawa

**Affiliations:** Department of Surgical Pathology, Kyoto Prefectural University of Medicine, 465 Kajii-cho, Kamigyo-ku, Kyoto 602-8566 Japan; Department of Surgery, Division of Digestive Surgery, Kyoto Prefectural University of Medicine, 465 Kajii-cho, Kamigyo-ku, Kyoto 602-8566 Japan

**Keywords:** Colorectal carcinoma, Poorly differentiated clusters, Semi-quantitative evaluation, Histological prognostic indicator

## Abstract

**Background:**

Poorly differentiated clusters (PDCs) at the invasive front of tumors in colorectal cancer (CRC) have recently been highlighted as histological prognosticators. We aimed to assess the clinical importance of extent of PDCs in CRC.

**Methods:**

A total of 239 patients with non-mucinous pT2 and pT3 CRC were pathologically reviewed. PDCs were defined as cancer clusters composed of ≥5 cancer cells lacking full glandular formation. Patients were classified according to the number of PDCs observed under a × 20 objective lens. Patients with <5 clusters were classified as G1, those with 5 to 9 clusters were classified as G2, and those with ≥10 clusters were classified as G3. In addition, in order to semi-quantitatively evaluate the PDCs, the extent of the highest grade of PDCs at the tumor’s invasive front was measured and summated, if separately distributed. We identified cutoffs for the extents of PDCs and compared the results with the patients’ survival rates.

**Results:**

The number of patients with G1, G2, and G3 clusters was 140, 46, and 53, respectively. The presence of G3 PDCs was significantly correlated with lymphatic permeation (*P* < 0.0001) and node involvement (*P* < 0.0001). The 5-year overall survival rates of G1, G2, and G3 were 91%, 88%, and 76%, respectively. Based on the Kaplan-Meier method, 5- and 10-mm cutoffs were identified as the statistically reliable stratification for the extents of G3 clusters, and 15, 20, and 18 G3 patients exhibited extents of <5 mm, 5 to 9 mm, and ≥10 mm, respectively; however, cutoffs for the extents of G1 and G2 clusters were not obtained. In the subgroup analysis, when the extents of G3 clusters were subclassified into <5 mm as G3a, 5 to 9 mm as G3b, and ≥10 mm as G3c, the 5-year overall survival rates were 83%, 62%, and 44%, respectively.

**Conclusions:**

G3 PDCs were highly indicative of tumor aggressiveness. Quantitative evaluation that takes into account the extent of PDCs would provide more concise prognostic information. Our results suggest that G3 PDCs with a larger extent are closely associated with unfavorable patient outcome.

## Background

The incidence of colorectal carcinoma (CRC) is on the rise worldwide, and the mortality and morbidity rates are high in both men and women [1]. Current therapeutic strategies for CRC are based on curative surgery and postoperative adjuvant chemotherapy. However, it has been reported that despite curative surgery, colorectal metastatic disease still leads to death in approximately 30% of cases [2]. As such, early identification of postoperative recurrent risk is critical for the determination of appropriate intervention strategies and for the improvement of patient outcomes.

Since Dukes [3] first reported the histological grading of rectal cancer in 1932, factors such as depth of tumor invasion, regional lymph node involvement, and remote metastasis have been universally accepted as clinical predictors of CRC [4,5]. Recently, Ueno *et al*. [6] were the first to describe poorly differentiated components or poorly differentiated clusters (PDCs) in CRC, and their clinical usefulness was also reported in patients with invasive breast cancer [7]. Thus, further examination of PDCs could bring a complementary approach to the pathological diagnostic process of CRC. PDCs are defined as cancer clusters composed of more than five cancer cells, present at the invasive front of a tumor, and lacking full glandular formation [6]. Although tumor dedifferentiation and dissociation have been histologically regarded as important first steps in tumor cell invasion [8], some recent reports documented that PDCs of CRC were closely associated with lymph node involvement and could provide a useful histological indication for tumor cell invasion [9-11]. However, only limited data concerning PDCs [9] have successfully clarified their impact on long-term patient outcomes; further validation is still needed. It has been well-established that histological intratumoral heterogeneity increases as a tumor progresses [12]. However, to date, the clinical significance of varying extents of PDCs - such as extensively distributed lower grade PDCs or locally contained higher grade PDCs - has not been fully investigated. Further understanding of PDCs’ extent would contribute to the improvement and standardization of the diagnosis of CRC. In the present study, we expanded upon the existing PDC scoring system [8] and assessed how the extents of PDCs are associated with the clinical outcome of CRC patients.

## Methods

### Patients

Data from consecutive patients with non-mucinous pT2 and pT3 CRC [4] who had undergone potentially curative resection and were enrolled from 2000 to 2005 were used. The exclusion criteria of this retrospective cohort were as follows: (1) synchronous or multiple cancers, (2) preoperative chemotherapy, (3) cancers related to ulcerative colitis or Crohn’s disease, and (4) familial adenomatous polyposis or hereditary non-polyposis colorectal cancer.

The sectioning procedure applied to resected specimens after formalin fixation was performed according to the Japanese guidelines [5]. In brief, each specimen was sectioned in a crosswise direction to include the central part of the tumor. When smaller tumors were examined, the specimen was step-sectioned at approximately 5-mm intervals parallel to the long axis of the intestinal tract. The slides were then stained using a conventional hematoxylin and eosin staining procedure. A total of three to four slides per specimen were obtained.

Written informed consent was obtained from all the patients prior to the initiation of this study, and this retrospective study was approved by the Institutional Review Board at the Kyoto Prefectural University of Medicine (ERB-C-41).

### Histopathological evaluation

**Figure 1:**
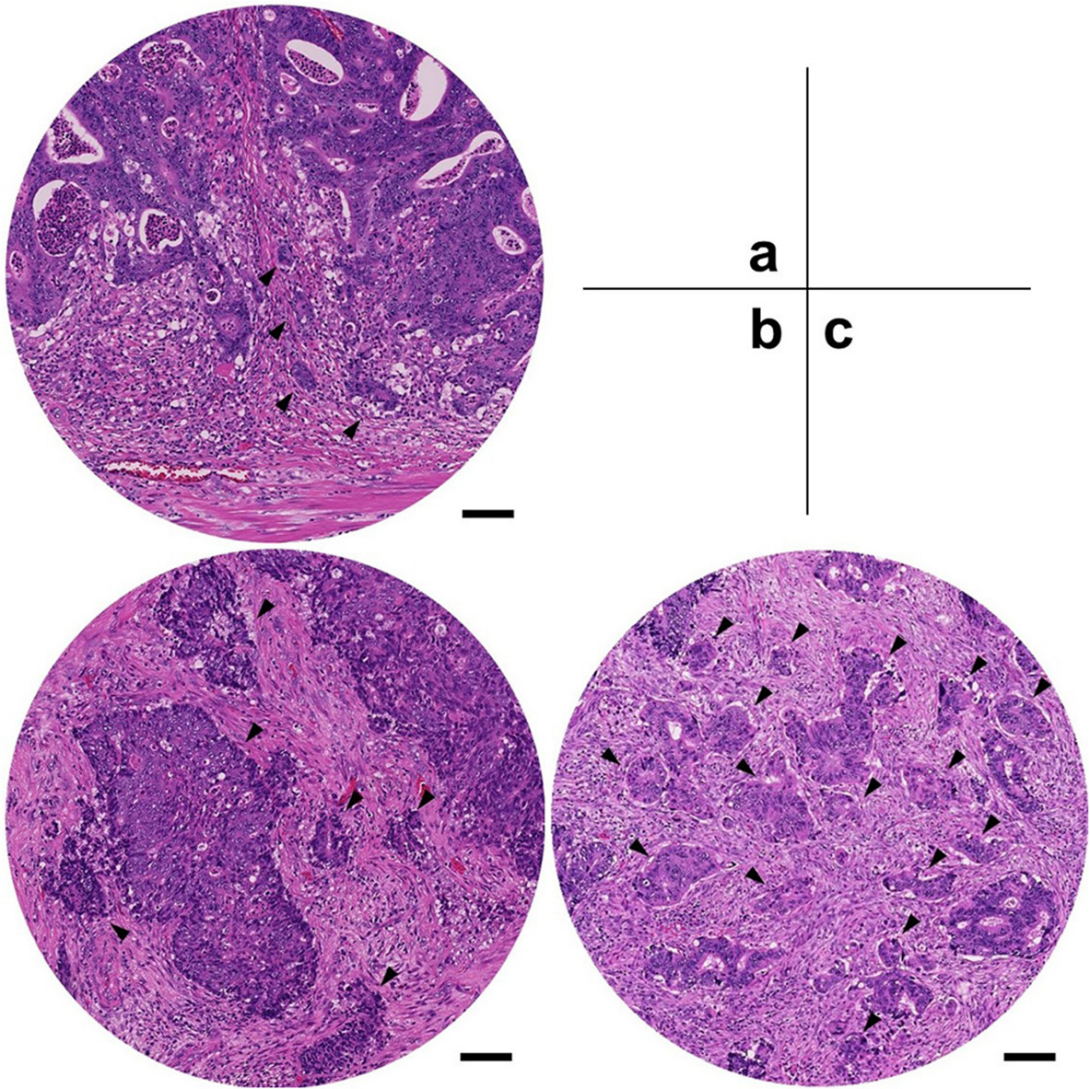
**Tumors assigned into three grades depending on the number of PDCs.** Arrowheads indicate poorly differentiated clusters (PDCs). **(a)** For G1, four clusters are observed; **(b)** for G2, six clusters are observed regardless of cluster size; and **(c)** for G3, more than ten clusters are observed under × 20 objective lens (0.950 mm^2^ field of vision). The scale bar indicates 100 micrometers.

**Figure 2:**
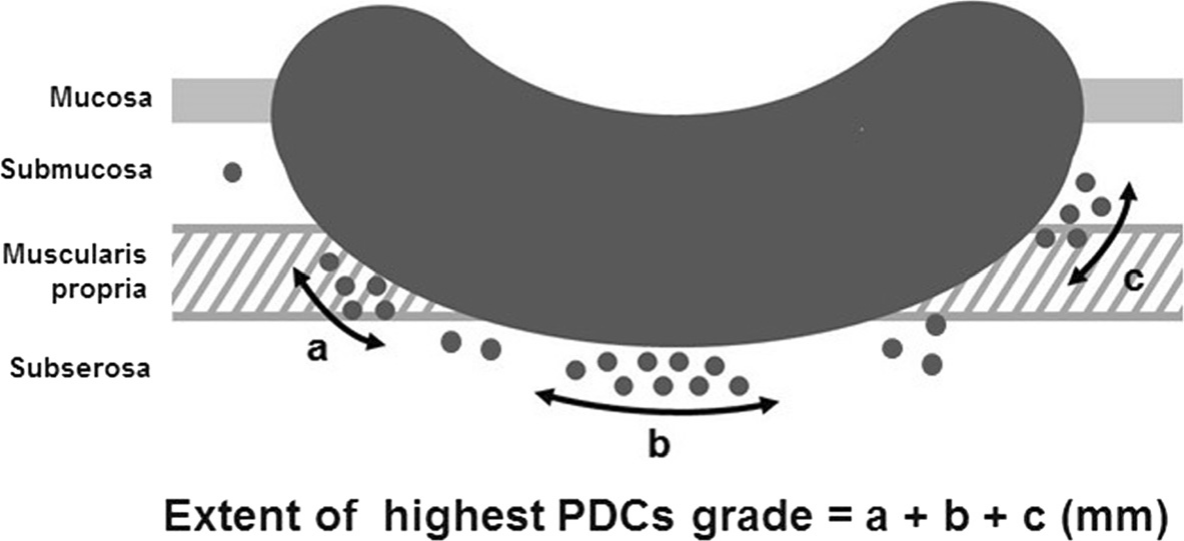
**Exemplification of measurement method.** PDCs: poorly differentiated clusters. For example, if the extents of the highest PDCs grade of ‘a’, ‘b’, and ‘c’ are distributed separately, they were summated as the final PDC extent for the analysis.

All slides were pathologically reviewed by a trained gastrointestinal-specialized pathologist. Based on previously set definitions [9], PDCs were defined as cancer clusters composed of ≥5 cancer cells lacking full glandular formation and identified at the invasive front of tumors. Tumors were assigned into three grades depending on the number of PDCs most commonly observed under a × 20 objective lens: G1 had <5 clusters; G2 had 5 to 9 clusters; and G3 had ≥10 clusters (Figure 1). Furthermore, we assessed the total extent of the highest PDC grade at the invasive front of the tumors in order to semi-quantitatively evaluate the significance of PDCs. In short, even when various grades of PDCs were observed within the same tumor, only the extent of the highest PDC grade was measured. When the highest PDC grade was separately distributed, the extents were summated (Figure 2). Finally, cutoffs for the extent of each PDC grade were identified, and a subgroup analysis was performed. For detection of lymphovascular invasion (LVI), D2-40 antibody (DAKO Cytomation, Glostrup, Denmark) and elastic staining were used for lymphatic permeation and vascular infiltration, respectively, according to the recommendations of the Japanese guidelines [5].

### Statistical analysis

Overall survival (OS) curves were plotted using the Kaplan-Meier method, and comparisons were performed using a log-rank test. The cutoff for the extents of the PDCs was optimized by maximizing the significance. A multivariate Cox proportional hazards regression analysis was used to assess the prognostic value of PDCs. A hazard radio (HR) and 95% confidence interval (CI) were determined using the Cox regression model. All statistical analyses were performed using Stat View 5.0 software (SAS Institute, Cary, NC, USA), and a *P* value of <0.05 was considered statistically significant.

## Results

**Figure 3:**
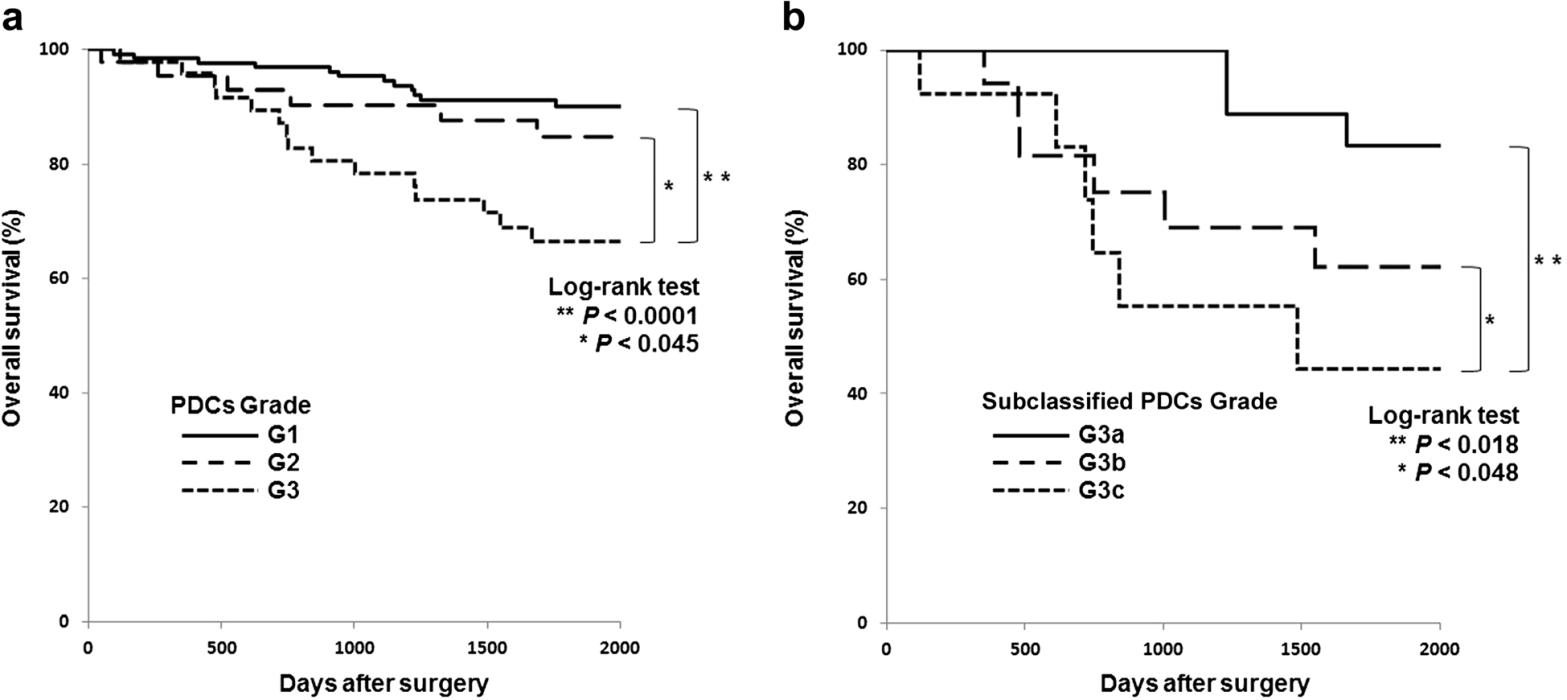
**Overall survival curves. (a)** Overall survival curves according to the highest poorly differentiated cluster (PDC) grade (left) and **(b)** according to subclassified G3 PDCs (right).

A total of 239 patients were eligible for pathological review. Patient characteristics are summarized in Table 1. The median follow-up period after surgery was 65.3 months (range, SD: 0.33 to 132.6, 34.5), and the 5-year OS rates were 91%, 87%, and 76% in stage I, stage II, and stage III, respectively. Since laparoscopic surgery was not widespread during the period of this study, operative procedures consisted of laparotomic interventions only. Based on the highest PDC grades observed in the tumor specimens, 140 patients (58.6%) were assigned as G1, 46 (19.2%) as G2, and 53 (22.2%) as G3. As shown in Table 2, G3 PDCs were significantly correlated with older age (≥65 years, *P* = 0.031), lymphatic permeation (*P* < 0.0001), and lymph node involvement (*P* < 0.0001). No significant differences were found in the clinicopathological factors between the colon and rectal carcinomas. When representative clinicopathological variables were evaluated by univariate analysis, lymph node involvement (*P* = 0.036) and G3 PDCs (*P* < 0.0001) were found to be significantly associated with short survival. As shown in Figure 3a, the 5-year OS rates of G1, G2, and G3 PDCs were 91%, 84%, and, 66%, respectively. Furthermore, multivariate analysis showed that G3 was the most reliable prognostic factor (Table 3).

**Table 1:** **Patient characteristics (** ***n*** **= 239)**

**Parameters**		**Value**
Sex (*n* (%))	Male	131 (55)
	Female	108 (45)
Age (mean (range, SD))		66.7 (23 to 87, 10.3)
Tumor location (*n* (%))	Colon	147 (62)
	Rectum	92 (38)
Depth (n (%))	pT2	63 (26)
	pT3	176 (74)
Lymph node involvement (*n* (%))	pN0	146 (61)
	pN1	75 (31)
	pN2	18 (8)
Total number of retrieved lymph nodes (mean (range, SD))		15.6 (0 to 61, 9.77)
Lymphatic permeation (n (%))	Positive	138 (58)
	Negative	101 (42)
Venous infiltration (*n* (%))	Positive	127 (53)
	Negative	112 (47)
Adjuvant chemotherapy (*n* (%))	With	119 (50)
	Without	96 (40)
	Unknown	24 (10)
PDCs grade (n (%))	G1	140 (59)
	G2	46 (19)
	G3	53 (22)

SD: standard deviation, PDC: poorly differentiated cluster.

**Table 2:** **Correlation between PDC grade and clinicopathological parameters**

**Parameters**		**PDC grade**			***P*** **value**
		**G1**	**G2**	**G3**	
Sex (*n* (%))	Male	75 (31)	28 (12)	28 (12)	0.65
	Female	65 (27)	18 (8)	25 (10)	
Age (*n* (%))	≥65	52 (22)	10 (4)	25 (10)	0.031
	<65	88 (37)	36 (15)	28 (12)	
Tumor location (*n* (%))	Colon	84 (35)	29 (12)	34 (15)	0.84
	Rectum	56 (23)	17 (7)	19 (8)	
Tumor diameter (mm) (mean)		42.0	44.2	44.3	
Depth (n (%))	pT2	43 (18)	10 (4)	10 (7)	0.26
	pT3	97 (40)	36 (15)	43 (18)	
Lymph node involvement (*n* (%))	pN0	98 (41)	28 (12)	20 (9)	<0.0001
	pN1	40 (17)	15 (6)	20 (8)	
	pN2	2 (1)	3 (1)	13 (5)	
Total number of retrieved lymph nodes (mean)		14.1	14.9	16.8	
Lymphatic permeation (n (%))	Positive	74 (31)	16 (7)	42 (17)	<0.0001
	Negative	66 (28)	30 (13)	11 (5)	
Venous infiltration (*n* (%))	Positive	73 (31)	19 (8)	19 (8)	0.095
	Negative	67 (28)	27 (11)	34 (14)	
Adjuvant chemotherapy (*n* (%))	With	63 (26)	27 (11)	29 (12)	0.22
	Without	63 (26)	14 (6)	19 (8)	
	Unknown	14 (6)	5 (2)	5 (2)	

PDC: poorly differentiated cluster.

**Table 3:** **Univariate and multivariate survival analyses of representative histopathological variables**

**Parameters**	**Univariate**	**Multivariate**		
		**Hazard ratio**	**95% CI**	***P*** **value**
Age (≥65 *vs*. <65)	0.55	1.02	0.54 to 1.91	0.9495
Tumor location (colon *vs*. rectum)	0.80	1.16	0.61 to 2.21	0.6517
Depth (pT3 *vs*. pT2)	0.38	1.19	0.56 to 2.53	0.6463
Lymph node involvement (pN1-2 *vs*. pN0)	0.036	1.35	0.70 to 2.60	0.3758
PDC grade (G3 *vs*. G1-2)	<0.0001	3.21	1.67 to 6.20	<0.0005

CI: confidence interval, PDC: poorly differentiated cluster.

**Figure 4:**
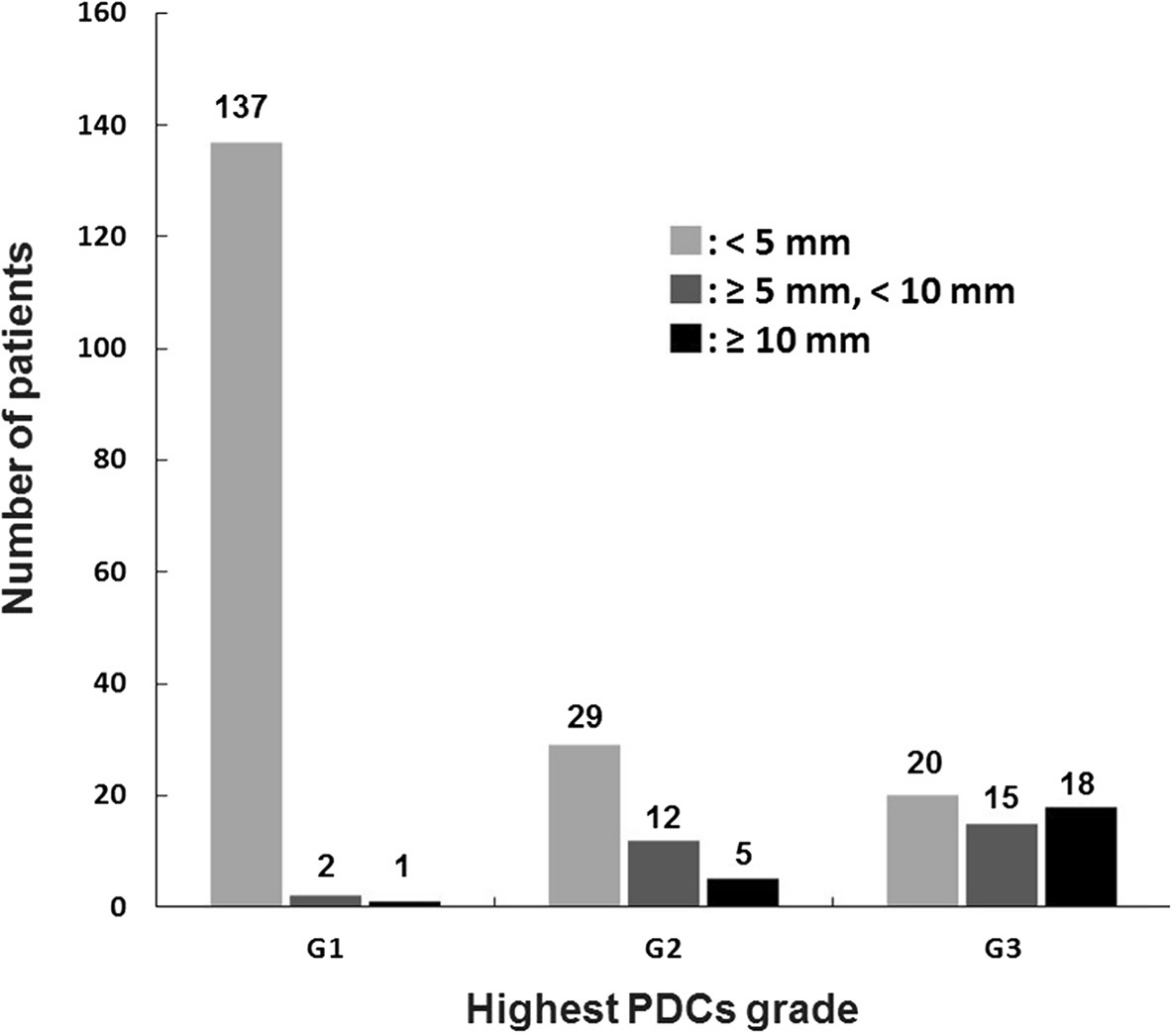
**Stratification of patients based on the extent of each poorly differentiated clusters (PDCs) in each grade.**

By using the Kaplan-Meier method for achieving the most statistically efficient discrimination for the patients’ prognoses, cutoffs of 5 mm (*P* = 0.018) and 10 mm (*P* = 0.048) for the extent of G3 PDCs were identified; however, no significant cutoffs for PDCs’ extents were obtained for G1 and G2 PDCs (Table 4). Figure 4 breaks down the stratification of the patients based on the extent of PDCs in each grade; 137, 2, and 1 G1 patients; 29, 12, and 5 G2 patients; and 15, 20, and 18 G3 patients exhibited extents of <5 mm, 5 to 9 mm and ≥10 mm, respectively. When the extent of G3 was subclassified into <5 mm as G3a, 5 to 9 mm as G3b, and ≥10 mm as G3c, subgroup analyses of G3 cases demonstrated that the 5-year OS rates of G3a, G3b, and G3c were 83%, 62%, and 44%, respectively (Figure 3b). Additionally, in the subgroup analysis, the patients who received adjuvant chemotherapy *vs*. those who did not were 10 *vs*. 5; 8 *vs*. 12; and 11 *vs*. 7 in G3a, G3b, and G3c, respectively. The 5-year survival rates were as follows: in G3a: 88% *vs*. 80%; in G3b: 64% *vs*. 60%; and in G3c: 42% *vs*. 49%.

**Table 4:** **Evaluation of the cutoff value for extent of G3 PDCs**

**Extent of G3 PDCs**	***P*** **value**
<10 mm *vs*. ≥10 mm	0.32
<9 mm *vs*. ≥9 mm	0.27
<8 mm *vs*. ≥8 mm	0.27
<7 mm *vs*. ≥7 mm	0.16
<6 mm *vs*. ≥6 mm	0.027
<5 mm *vs*. ≥5 mm	0.0043
<4 mm *vs*. ≥4 mm	0.018
<3 mm *vs*. ≥3 mm	0.019
<2 mm *vs*. ≥2 mm	0.035
<1 mm *vs*. ≥1 mm	0.16

PDCs: poorly differentiated clusters.

## Discussion

In the present study, we pathologically examined 239 consecutive patients with non-mucinous pT2 and pT3 CRC in order to (1) validate the clinical importance of PDCs based on a single institution experience and (2) demonstrate the extent of PDCs and determine its diagnostic importance. To our knowledge, no previous report has documented the diagnostic significance of extent of PDCs. In this study, we used a highly selective cohort of CRC. Although previous reports suggested that mucinous carcinoma may exhibit similar oncological aspects as PDCs [6,13], researchers have not yet reached a consensus regarding their biological similarities. Therefore, mucinous carcinomas were excluded from this study.

Our results showed that the presence of G3 PDCs was significantly correlated with lymphatic permeation and lymph node involvement and was an unfavorable histological indicator. These results are in line with prior reports [6,9-11]. With regard to the assignment of the highest grade of PDCs, Ueno *et al*. [9] classified 50%, 30%, and 20% among a total of 3,242 patients with stages I to III CRC as G1, G2, and G3 PDCs, respectively. In this study, 59%, 19%, and 22% of patients with pT2-3 non-mucinous CRC were classified as G1, G2, and G3 PDCs, respectively. Our results were comparable to the aforementioned findings.

Previous methodologies have provided primarily qualitative or limited quantitative information, which does not aptly reflect the extent of PDCs. In this study, we aimed to clarify the prognostic value of the extents of PDCs. Our results demonstrated that the extents of G1 or G2 are not strong prognosticators, as we did not obtain any significant cutoff values for them. That is, even when G1 and G2 PDCs predominantly distribute, quantitative evaluation of these PDCs cannot provide any valuable prognostic information. On the other hand, since our analyses showed a statistically significant cutoff extent for G3, special consideration should be given to the extents of G3 PDCs during the diagnostic process. In other words, G3 PDCs with larger extents are predictive of a very poor survival outcome, whereas the 5-year OS rate of patients with <5-mm extents of G3 PDCs (83%) was close to that of G2 patients (88%). These results suggest that the extents of G3 PDCs could have a potential impact on patient survival.

This study has a number of limitations. First, because this was a retrospective cohort study, there was no control over the type of adjuvant chemotherapy given to patients, its course, or its completion. However, this study was also designed to investigate the indication for adjuvant chemotherapy; as a result, half of the patients chosen for this study received adjuvant chemotherapy. In the subgroup analysis of G3 PDCs in which the patients with and without adjuvant chemotherapy were compared, no significant differences were found in the 5-year survival rates of the two. A previous report [6] described similar findings concerning the correlation between adjuvant chemotherapy and PDCs. Developing effective adjuvant chemotherapy is challenging and merits further studies with larger sample sizes. The second limitation is that lymphatic permeation (58%) and vascular infiltration (53%) may have been overestimated in this group of patients. Although the reason for this is uncertain, a possible explanation is that the higher detection rate may have been the result of using multiple slides for evaluation combined with the routine use of D2-40 antibody and elastic staining. Many authors have reported that endothelial markers may help the detection of LVI; however, interobserver concordance remains low, even among gastrointestinal-specialized pathologists [14,15].

## Conclusions

In conclusion, PDCs are highly indicative of tumor aggressiveness in non-mucinous pT2 and pT3 CRC. A more quantitative diagnostic approach to the evaluation of PDCs which takes into account the extent of PDCs would provide more concise prognostic information. Overall, our results suggest that the extents of G3 PDCs require special attention during diagnosis and that G3 PDCs with larger extents are closely associated with unfavorable patient outcomes.

## Abbreviations

CI: confidence interval
CRC: colorectal carcinoma
HR: hazard radio
LVI: lymphovascular invasion
OS: overall survival
PDCs: poorly differentiated clusters
SD: standard deviation
